# A spontaneous heterotopic pregnancy presenting with acute abdomen treated with natural orifice transluminal endoscopic surgery procedure: Case report

**DOI:** 10.4274/tjod.galenos.2019.76300

**Published:** 2020-02-28

**Authors:** Keziban Doğan, Cihan Kaya, Derya Ece Ilıman, İbrahim Karaca, Hüseyin Cengiz

**Affiliations:** 1 University of Health Sciences, Bakırköy Dr. Sadi Konuk Training and Research Hospital, Clinic of Obstetrics and Gynecology, İstanbul, Turkey

**Keywords:** Heterotopic pregnancy, acute abdomen, natural orifice transluminal endoscopic surgery procedure

## Abstract

Heterotopic pregnancy occurs 1 in 30000 pregnancies. We present case of an acute abdomen caused by a ruptured ectopic component. Our patient had no known risk factors, which made the diagnosis even more challenging. Intrauterine pregnancy was desired by patient and her husband. A natural orifice transluminal endoscopic surgery (NOTES) procedure was performed, which is a next-generation minimally invasive procedure in this area. After the procedure, our patient was discharged one day after surgery with a viable intrauterine pregnancy.

**PRECIS:** Heterotopic pregnancy treated with vNOTES procedure.

## Introduction

Heterotopic pregnancy is defined as the co-existence of an ectopic pregnancy and an intrauterine pregnancy. This condition occurs in 1 out of 30000 pregnancies of natural conception^(1)^. Assisted reproductive technologies have a higher rate of 9 out of 10000^(2)^. A previous history of pelvic inflammatory disease and pelvic surgery is associated with the incidence of heterotopic interstitial pregnancy^(3)^. Although ectopic pregnancies can be positioned at various anatomic locations such as cervix, ovary, and abdomen, tubal pregnancies have the highest rate of approximately 95-96%^(4)^. In order to preserve the intrauterine pregnancy, certain clinical manifestations and diagnostic tests alternates compared to ectopic pregnancy.

Transvaginal natural orifice transluminal endoscopic surgery (vNOTES) is a less invasive alternative to laparoscopic salpingectomy. Abdominal cavity is reached through a port placed in the posterior fornix. The safety and efficacy of transvaginal endoscopic salpingectomy for tubal ectopic pregnancy are equivalent to those of laparoscopic procedures. Lesser postoperative pain and a more satisfactory cosmetic outcome were found with the transvaginal endoscopic procedure, making it the more preferred method and superior to the laparoscopic approach^(5)^.

## Case Report

**Figure 1:**
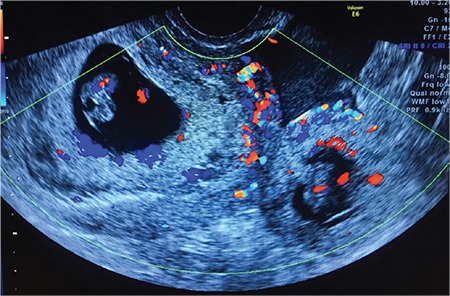
Ultrasound of intrauterine pregnancy and ectopic pregnancy with cardiac activity on Doppler

**Figure 2:**
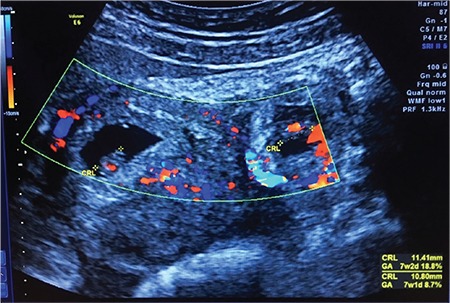
Twin heterotopic pregnancy with estimated gestational age of 7 weeks and 1 day and 7 weeks and 2 days

This report presents a 29-year-old G1P0 patient who presented to the obstetrics emergency room with severe abdominal pain of the left lower quadrant with signs of acute abdomen and knowledge of a 7 weeks’ intrauterine pregnancy. The patient was evaluated by the obstetric team upon arrival and her heart rate was 99 bpm, and blood pressure was 90/50 mm Hg. A transvaginal ultrasound was performed, which revealed a 7 weeks and 2 days estimated gestational age intrauterine pregnancy with fetal cardiac activity and was considered viable. Both of the patient’s fallopian tubes were dilated and showed evidence of a hydropic fallopian tube, which was evaluated as evidence of former pelvic inflammatory disease. Additionally, a 10.8-cm ectopic pregnancy was positioned on the left side with cardiac activity with an estimated gestational age of 7 weeks and 1 day. Blood products and abdominal free fluid were also detected. At the time, the patient’s hemoglobin and hematocrit levels were 12.2 g/dL and 35.4% respectively, and the other initial blood parameters checked were within the normal range (Figure 1, 2).

### Assessment

The patient’s condition was stabilized with a saline infusion and informed about her clinical condition. The patient expressed a desire to preserve the intrauterine pregnancy. Under close monitoring, the patient was admitted to the gynecology department and was given full information about her condition including the risk of miscarriage and more extensive surgery. After giving consent for the recommended surgery, the patient was taken to the operating room.

The patient was placed in the lithotomy position under general anesthesia. Following the sterile covering of the area, posterior colpotomy was used to enter the abdomen. Approximately 200 cc of blood containing clots was drained. The right and left ovary and right fallopian tube were observed in normal anatomic position and nature. The left tube was visualized, which had an approximately 5 cm ectopic mass with active bleeding. After visualizing using a camera, salpingectomy was performed on the left tube using a bipolar energy device. Afterwards, the pelvic area was examined and no bleeding was seen. The colpotomy was closed using sutures. There were no complications.

Follow-up ultrasound was performed in the post-operative 9^th^ hour. The intrauterine pregnancy was detected as viable. Two hundred milligrams of natural progesterone was ordered by vaginal route once the pregnancy was confirmed as viable. On postoperative day 1, the patient’s clinical condition and hemoglobin levels were stable and she was discharged. The patient was told to come for a follow-up examination.

One week after discharge, she presented for postoperative follow-ups and screening of the intrauterine pregnancy. Ultrasound showed a live fetus at 8-weeks 0-day gestation based on crown-rump length.

## Discussion

The patient who was admitted to our emergency department with acute abdomen and was diagnosed as having heterotopic pregnancy. She underwent salpingectomy with vNOTES and was discharged in good health. Although it is very challenging to diagnose heterotopic pregnancy, high-resolution transvaginal ultrasound is helpful in the process. Yet 20-50% of patients with an interstitial pregnancy present with rupture of the ectopic mass^(6)^. There are several options for the treatment of heterotopic pregnancy; surgical, medical or expectant treatment. The patient can be treated surgically through salpingectomy or hysterectomy either by laparotomy or laparoscopy. Another option is the direct injection of potassium chloride, hypertonic solution, and methotrexate into the ectopic gestational sac^(3,7,8)^. Lastly, if the patient has no symptoms and fetal death in gestation is confirmed using ultrasonography, expectant management can be used^(9)^. The other new way for surgical treatment is vNOTES.

VNOTES is considered to be a next-generation minimally invasive surgical procedure; thus, numerous efforts in this area are being made in many countries. Recently, vNOTES has been performed for cholecystectomy, appendectomy, nephrectomy, and several gynecologic procedures^(10,11,12,13)^. Although NOTES procedure can be performed through various natural orifices, gynecologists are familiar with the vaginal area; therefore, it has gained more popularity than transanal or transgastric procedures. vNOTES had been performed successfully in a series of surgical procedures including salpingectomy, ovarian cystectomy, myomectomy, hysterectomy, lymphadenectomy, and sacrocolpopexy. Advances in technology have improved the feasibility of vNOTES as a treatment option for gynecologic surgeries. The advantages of vNOTES include reduced postoperative pain, faster post-operative recovery, and decreased postoperative wound infections, as well as outstanding cosmetic results^(14)^. Access through posterior colpotomy can be challenging if the patient has an adnexal mass or any adhesions causing cul-de-sac obliteration. It is advised to perform a careful vaginal examination before performing this procedure^(13)^. vNOTES salpingectomy, which can be performed successfully in women diagnosed with ectopic pregnancy, can be used safely in heterotopic pregnancy as seen in our case^(15,16)^. Although heterotopic pregnancy is quite rare, it should always be in the differential diagnosis of acute abdomen of pregnant patients.
